# Increased expression of the retinoic acid-metabolizing enzyme CYP26A1 during the progression of cervical squamous neoplasia and head and neck cancer

**DOI:** 10.1186/1756-0500-7-697

**Published:** 2014-10-07

**Authors:** Makoto Osanai, Gang-Hong Lee

**Affiliations:** Department of Pathology, Kochi University School of Medicine, Kohasu, Oko-cho, Nankoku, Kochi, 783-8505 Japan

**Keywords:** Retinoic acid, Retinoic acid-metabolizing enzyme CYP26A1, Vitamin A deficiency, Carcinogenesis, Cervical squamous neoplasia, Squamous intraepithelial lesion, Squamous cell carcinoma, Head and neck cancer, Immunohistochemistry

## Abstract

**Background:**

Retinoic acid (RA) is a critical regulator of cell differentiation, proliferation, and apoptosis in various cell types. Recently, the RA-metabolizing enzyme CYP26A1 (cytochrome P450, family 26, subfamily A, polypeptide 1) has been shown to have an oncogenic function in breast carcinogenesis. However, the relevance of elevated CYP26A1 expression in human cancers remains to be clarified.

**Methods:**

We immunohistochemically examined the expression of CYP26A1 in cervical squamous cell carcinoma (SCC) and its precursors, including low- and high-grade squamous intraepithelial lesions (LSIL and HSIL, respectively), as well as head and neck cancer (HNC). The association between CYP26A1 expression and a number of clinicopathological parameters was also evaluated.

**Results:**

CYP26A1 was not expressed in normal cervical epithelium. CYP26A1 expression was present in LSIL but limited to basal and parabasal cells. HSIL cases exhibited strong nuclear expression of CYP26A1 and mixed cytoplasmic expression patterns with widely distributed expression toward the epithelial surface. Importantly, strong cytoplasmic staining of CYP26A1 was observed in 19 of 50 (38%) patients with cervical SCC. Elevated expression of CYP26A1 was significantly associated with younger age (<50 years) and lymph node involvement (pN). Similarly, CYP26A1 was not expressed in non-neoplastic tissues of the head and neck, but strong cytoplasmic staining of CYP26A1 was observed in 52 of 128 (41%) HNC cases. Such strong CYP26A1 expression was significantly associated with the primary tumor stage of carcinomas (pT) and the pathological tumor-node-metastasis (pTNM) stage in HNC.

**Conclusion:**

Our results indicated an elevated CYP26A1 expression in malignant and precancerous dysplastic lesions of the human cervix, which also increased with the progression of cervical squamous neoplasia. In addition, this report is the first to demonstrate the increased expression of CYP26A1 in HNC and its significant correlation with primary tumor growth. These data suggested that CYP26A1 overexpression might contribute to the development and progression of cervical malignancies and squamous neoplasia of the head and neck.

**Electronic supplementary material:**

The online version of this article (doi:10.1186/1756-0500-7-697) contains supplementary material, which is available to authorized users.

## Background

Cervical carcinoma is the second most common cancer in women worldwide [1]. This malignant neoplasm develops from basal cells originating in the uterine cervix, and squamous cell carcinoma (SCC) is the most common presentation of cervical cancer, accounting for approximately 80–85% of all cases [1]. Chronic infection with human papilloma virus (HPV), especially the high-risk variants HPV16 and 18, is the single most important etiologic factor in the pathogenesis of cervical carcinoma and its precursors [1, 2]. Multiple risk factors, such as sexual activity at an early age, high parity, smoking, and use of oral contraceptives, are associated with cervical carcinoma. An emerging body of evidence suggests that poor nutritional status is also a risk factor for the malignant transformation of cervical cells [3]. Additionally, improvement in nutrition status may play a role in the prevention and reversal of cervical dysplasia [4]. Reversal of mild and moderate cervical dysplasia has been demonstrated in patients receiving vitamin A supplementation over a few month period [5]. Furthermore, a number of clinical studies have shown that topical application of a vitamin A derivative to the cervix completely reverses cervical dysplasia in 50% of cases [6].

Head and neck cancer (HNC) is the sixth most common human malignancy, accounting for approximately 3% of all cancer types with a 50% mortality rate [7, 8]. It consists of biologically similar tumors that arise from the squamous mucosa of the oral cavity, nasal cavity, lips, larynx, and pharynx. Approximately 90% of these tumors are SCC. HNC is closely associated with a number of environmental risk factors, including tobacco smoking, alcohol consumption, and infection with certain strains of virus, such as HPV and human herpes virus [9, 10]. The carcinogenesis of HNC is a multifactorial process involving a wide array of genetic events that alter the normal functions of oncogenes and tumor suppressor genes [11, 12]. However, the exact molecular mechanisms that govern the onset and development of HNC remain to be clarified.

Retinoic acid (RA), an active metabolite of vitamin A, is a critical signaling molecule involved in the differentiation, proliferation, and apoptosis of a wide variety of cell types. Recently, the RA metabolizing enzyme CYP26A1 (cytochrome P450, family 26, subfamily A, polypeptide 1) has been shown to promote the survival and oncogenic potential of breast carcinoma cells, indicating a possible oncogenic function of CYP26A1 in breast carcinogenesis [13]. Indeed, enhanced RA metabolism and elevated CYP26A1 levels have been observed in various types of cancer [14–16]. These observations are consistent with the accumulated evidence on the association of vitamin A deficiency (VAD) with increased susceptibility to carcinogenesis and elevated risk for a number of human cancers [17].

To date, the relevance of elevated CYP26A1 expression in human cancers has yet to be clarified. Therefore, the present investigation aimed to examine the possible association between CYP26A1 expression and squamous neoplastic development or progression via immunohistochemical studies. We determined CYP26A1 expression in non-neoplastic (normal) cervical epithelium, precancerous cervical dysplastic lesions, including low- and high-grade squamous intraepithelial lesions (LSIL and HSIL, respectively), and SCC. We also examined CYP26A1 expression in HNC using tissue microarray slides. In addition, the association between CYP26A1 expression and a number of clinicopathological factors was also evaluated for both cervical carcinoma and HNC.

## Methods

### Patient samples

To examine CYP26A1 expression in cervical neoplasia, 52 archived formalin-fixed, paraffin-embedded tissue specimens prepared from surgically resected materials and biopsy samples were studied (Additional file 1: Table S1). An experienced surgical pathologist evaluated hematoxylin-eosin stained slides of all specimens and CYP26A1 expressions by immunohistochemistry. Informed consent was obtained from all patients for pathological assessment of their specimens, and the Ethics Committee of Kochi University School of Medicine approved the present study. Cervical neoplasias were classified according to histological types using the World Health Organization guideline [1] and the Bethesda system [18].

### Tissue samples

Tissue sections and tissue microarray slides (Super Bio Chips, Seoul, Korea) were used for cervical carcinoma studies. In addition, two different tissue microarray slides were utilized for HNC studies, including the multiple head and neck cancer microarray (US Biomax, Rockville, MD, USA) and the human laryngeal and pharyngeal cancer microarray (Super Bio Chips). The clinicopathological variables examined included age, sex, primary tumor status (pT), lymph node involvement (pN), distant metastasis (M), and pathological tumor-node-metastasis (pTNM) stage. Cases with inadequate or inapplicable data for each category as provided by the manufacturers were excluded from the statistical analysis.

### Immunohistochemistry

Tissue sections and tissue microarray slides were deparaffinized in xylene, rehydrated through a graded series of ethanol and phosphate-buffered saline, and incubated in 3% H_2_O_2_ for 10 minutes to block endogenous peroxidase activity. After antigen retrieval by microwave heating (500 W) in citrate buffer for 15 minutes, the sections were incubated overnight at 4°C with a primary monoclonal antibody against CYP26A1 (1:50; clone F27 P6 A1, Santa Cruz Biotechnology, Santa Cruz, CA, USA). The sections were then incubated with EnVision (Dako, Glostrup, Denmark) for 30 minutes at room temperature, and color was developed using 3,3′-diaminobenzidine tetrachloride (Sigma, St. Louis, MO, USA) as the chromogen. The slides were subsequently counterstained with Meyer’s hematoxylin. Appropriate positive and negative controls were used in each experiment, and the results were confirmed by independent duplicate assays.

For cervical carcinoma, immunohistochemical staining was scored according to the percentage of positively stained tumor cells as follows: 3+ (strong), >50% positive cells; 2+ (moderate), 25–49% positive cells; 1+ (weak), 5–24% positive cells, and 0 (negative), no stained cells or <5% positive cells. A score of 2+ or 3+ was considered positive for CYP26A1 expression, whereas a score of 0 or 1+ was considered negative.

For HNC, staining positivity was also semi-quantitatively analyzed, considering staining intensity and percentage of positive cells, owing to the heterogeneous staining pattern in tumors of HNC tissues. A score was assigned based on the percentage of positively stained tumor cells (the proportion score) as follows: 3+, staining in >50% of the cells; 2+, staining in 25–49% of the cells; 1+, staining in 5–24% of the cells, and 0, no or faint staining in <5% of the cells. In addition, another score was determined based on the immunoreactivity intensity (the intensity score) as follows: 3+, strong; 2+, moderate; 1+, weak, and 0, negative. The final score was the summation of the proportion and intensity scores and categorized as follows: 5–6, strongly positive; 3–4, moderately positive, and 0–2, weakly positive or negative. CYP26A1 expression was considered positive when the final score was ≥3 and negative with a score of ≤2.

### Quantification of immunohistochemical staining

Quantitative analysis of CYP26A1 expression was performed using histological images that were digitally obtained using a light microscope in order to best reflect the overall immunostaining of the specimen on each slide. A camera control with constant setup conditions was maintained throughout the course of the study. Adobe Photoshop software (Adobe Systems, Mountain View, CA, USA) was used to process acquired images. For quantification, brown areas (positive signals) were selected in each image and converted to grayscale. The non-specific chromogen signals were removed by binarizing each image setting at the appropriate threshold. The grayscale image was then converted to a binary form by changing the positive pixels to black and the negative pixels to white according to the threshold value. For each binary image, the area of positive regions was measured using Image J software (National Institutes of Health, Bethesda, MD, USA) and expressed as percent positivity relative to the entire image field. For each section, CYP26A1 quantification was verified in 2 separate arbitrarily selected fields. The positive CYP26A1 signals were scored in both fields, and the mean value was calculated.

### Statistical analysis

All data were obtained from independent duplicate experiments. Statistical differences were analyzed using the Pearson chi-square (χ^2^) test to assess the correlation between CYP26A1 expression and a number of clinicopathological parameters. A *P* value of <0.05 was considered statistically significant.

## Results

### Expression and localization of CYP26A1 in cervical intraepithelial neoplasia

**Figure 1:**
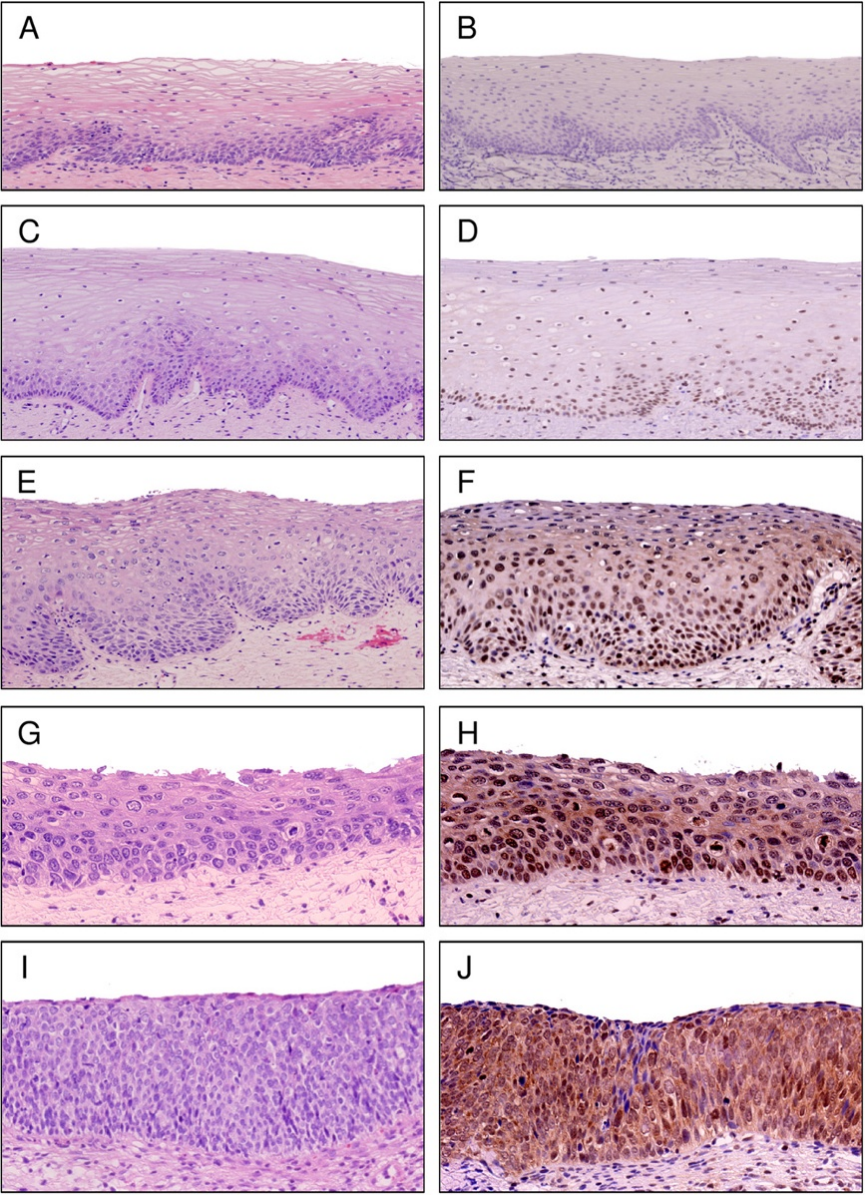
**CYP26A1 expression in cervical squamous intraepithelial neoplasia.** Representative images of hematoxylin-eosin staining **(A, C, E, G, and I)** and immunohistochemistry of CYP26A1 **(B, D, F, H, and J)**. CYP26A1 expression was evaluated in non-neoplastic (normal) squamous epithelium **(A, B)**, epithelium with mild **(C, D)**, moderate **(E, F)**, and severe **(G, J)** dysplasia, as well as squamous cell carcinoma *in situ* **(I, J)**. Original magnification: ×40 **(A, B, C, D, and E)**, ×100 **(F, G, H, I, and J)**.

We first examined the expression and distribution of CYP26A1 in cervical intraepithelial neoplasia (Figure 1, Additional file 1: Table S1). CYP26A1 expression was not detected in normal cervical tissues. Constitutive expression of CYP26A1 was observed in LSIL (n = 14), but it was restricted to basal and parabasal cells, as well as in the cells with koilocytosis. CYP26A1 was strongly expressed in all HSIL samples (n = 38), particularly in dysplasia with atypical cells occupying more than two-third of the epithelium thickness. In such cases, CYP26A1 positive signals were detected in proliferating dysplastic cells. Lesions with marked histological atypia and dysplastic changes showed stronger nuclear expression of CYP26A1 and a wide distribution of positive signals toward the epithelial surface. Although various mixed patterns of cytoplasmic staining were observed in HSIL cases, positive staining of CYP26A1 was detected only in the nuclei in LSIL cases.

### Quantification of CYP26A1 expression in cervical neoplasia

**Figure 2:**
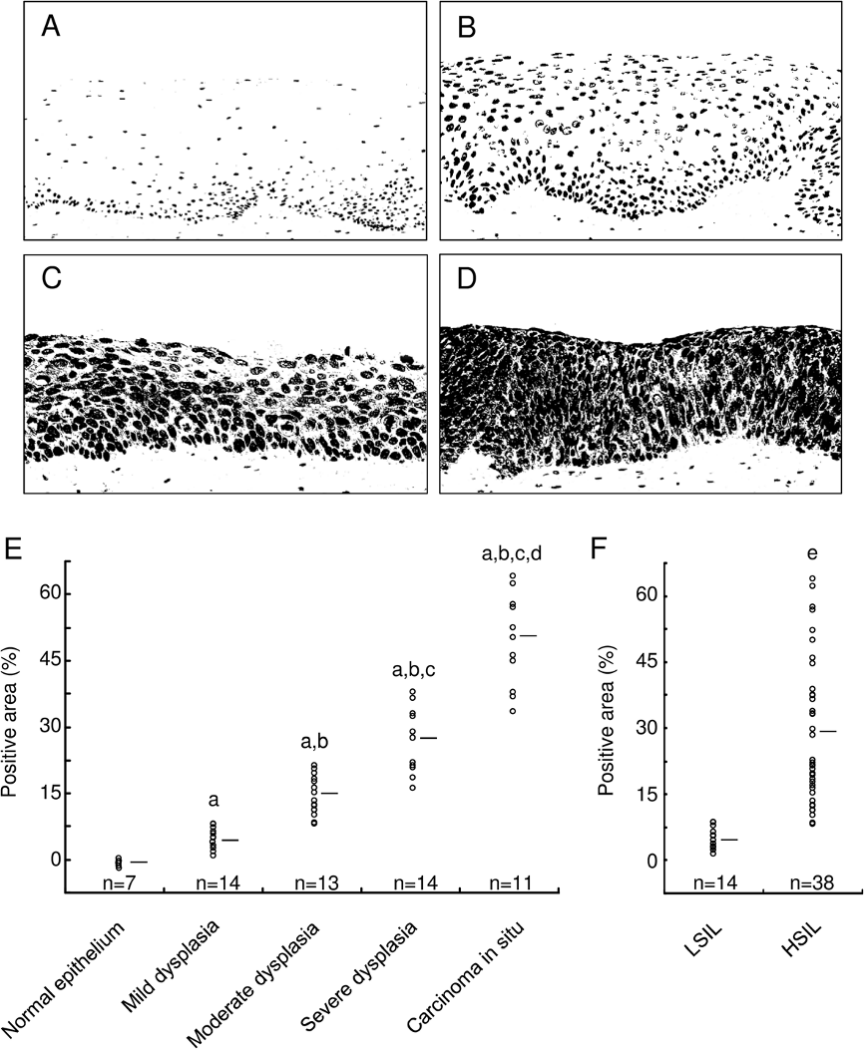
**Quantification of CYP26A1 expression in cervical squamous intraepithelial neoplasia. (A–D)** The color images of each representative field (e.g., Figure 1D, F, H, and J) were transformed into grayscale images and subsequently converted to binary images according to the threshold value. **(E and F)** Quantification of positive signals. Horizontal bars represent the mean values. *P* < 0.05 versus quantification of stained tissues from normal epithelium (a) or epithelium with mild (b), moderate (c), and severe (d) dysplasia, as well as those from low-grade squamous intraepithelial lesions (e).

We performed a semi-quantitative analysis of CYP26A1 expression in normal cervical epithelium and cervical intraepithelial neoplastic lesions by evaluating the area of positive signals per unit area of the entire tissue section (Figure 2). CYP26A1 positive signals were observed in cervical dysplasia, and the epithelium with dysplastic changes demonstrated more positive areas and signals. Importantly, HSIL cases with strong histological atypia were significantly highlighted by positive signals compared to LSIL cases (27.8 ± 16.0% versus 4.6 ± 1.6%; *P* < 0.05).

### Expression of CYP26A1 in cervical malignancies

**Figure 3:**
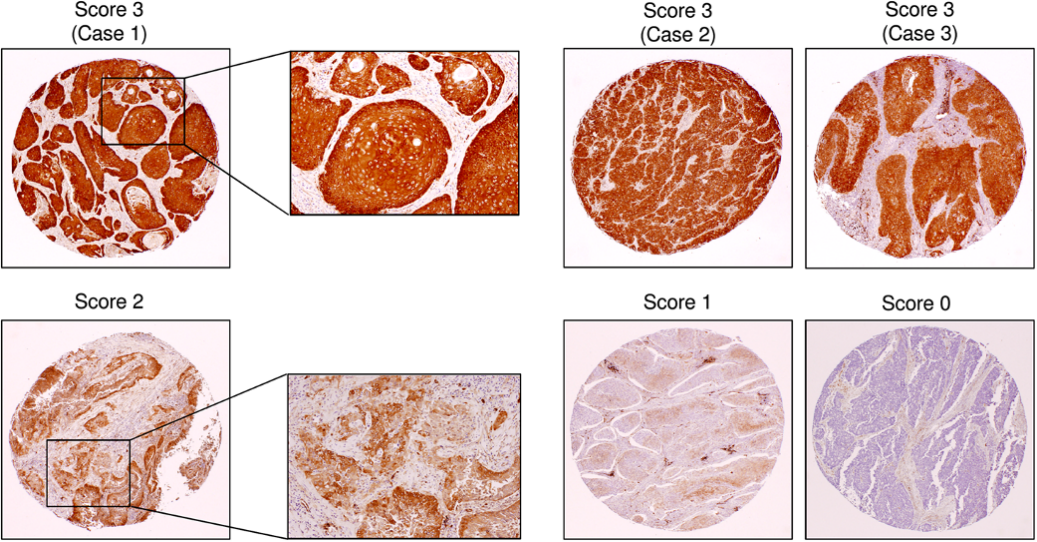
**CYP26A1 expression in cervical malignancies via tissue microarray analysis.** Representative cases of squamous cell carcinoma of the cervix for each CYP26A1 staining score are shown. Note the 3 representative cases with strong CYP26A1 positivity (staining score of +3). Original magnification: ×10; insets: ×200.

To examine the expression of CYP26A1 in cervical malignancies, tissue microarray analysis was performed (Figure 3, Additional file 2: Table S2). A strong diffuse cytoplasmic expression of CYP26A1 was observed in 19 of 50 (38%) SCC cases. In addition, strong expression of CYP26A1 was significantly associated with younger age (<50 years) and lymph node involvement (Table 1). In contrast, CYP26A1 immunoreactivity was not associated with Ki-67 labeling index, p53 expression, primary tumor status, or pTNM stage. Kaplan-Meyer analysis also failed to demonstrate a positive relationship between enhanced CYP26A1 expression and overall survival (data not shown).

**Table 1:** **CYP26A1 expression and the clinical profiles of the cervical cancer tissue microarray**

		CYP26A1 expression		
Variables	Cases (n)	Negative (%)	Positive (%)	***P***
Age (years)				
<50	21	9 (42.9)	12 (57.1)	0.022*
≥50	29	22 (75.9)	7 (24.1)	
Primary tumor status (pT)				
pTis	3	2 (66.7)	1 (33.3)	0.076
pT1a	7	7 (100)	0 (0)	
pT1b	35	18 (51.4)	17 (48.6)	
pT2	5	4 (80.0)	1 (20.0)	
Lymph node involvement (pN)				
Absent	31	24 (77.4)	7 (22.6)	0.016*
Present	19	8 (42.1)	11 (57.9)	
pTNM stage				
pStage 0	3	2 (66.7)	1 (33.3)	0.061
pStage IA	7	7 (100)	0 (0)	
pStage IB	20	13 (65.0)	7 (35.0)	
pStage II	1	1 (100)	0 (0)	
pStage III	19	8 (42.1)	11 (57.9)	
p53 expression				
Absent	44	25 (56.8)	19 (43.2)	0.071
Present	6	6 (100)	0 (0)	
Ki-67 labeling index				
<20%	35	25 (71.4)	10 (28.6)	0.056
≥20%	15	6 (40.0)	9 (60.0)	

*Significantly different by the chi-square (χ^2^) test.

*Abbreviations*: *CYP26A1*, cytochrome P450, family 26, subfamily A, polypeptide 1; *pTNM*, pathological tumor-node-metastasis

### Expression of CYP26A1 in HNC

**Figure 4:**
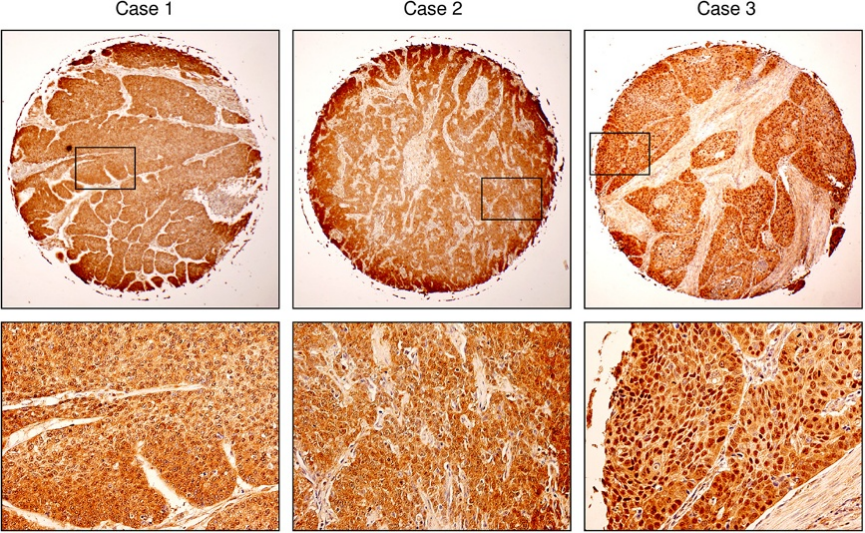
**CYP26A1 expression in head and neck carcinomas via tissue microarray analysis.** CYP26A1 overexpression in primary carcinomas of the head and neck was demonstrated using 3 representative cases with strong CYP26A1 staining. The lower panel (×200) is a magnified view of the rectangular area in the upper panel image (×10) for each case.

Previous studies have reported elevated levels of CYP26A1 in a wide variety of cancers [14–16]. We also observed a strong diffuse cytoplasmic expression of CYP26A1 in 52 of 128 (41%) HNC cases (Figure 4, Additional file 3: Table S3 and Additional file 4: Table S4). In contrast, no CYP26A1 immunoreactivity was detected in the normal mucosa, i.e., non-neoplastic squamous epithelium, of the head and neck. Furthermore, CYP26A1 overexpression was significantly associated with the primary tumor stage of carcinomas and pTNM stage (Table 2), suggesting that CYP26A1 was primarily correlated with primary tumor growth in HNC patients. Such an observation was consistent with our previously published results demonstrating that the enhanced expression of CYP26A1 had an oncogenic function in carcinogenesis [13]. On the other hand, the strong expression of CYP26A1 was an independent indicator of several variables, including age, sex, and lymph node involvement. Nonetheless, Kaplan-Meyer analysis failed to demonstrate a positive relationship between the enhanced expression of CYP26A1 and poor overall survival (data not shown). Interestingly, the strong nuclear expression of CYP26A1 was also observed in 18 of 128 (14%) HNC cases (Figure 4); however, no correlation was found between nuclear CYP26A1 positivity and any clinicopathological parameters.

**Table 2:** **Correlation between CYP26A1 expression and the clinicopathological variables in head and neck squamous cell carcinoma patients via tissue microarray analysis**

		CYP26A1 expression		
Variables	Cases (n)	Negative (%)	Positive (%)	***P***
Age (years)				
<60	59	31 (52.5)	28 (47.5)	0.136
≥60	61	41 (67.2)	20 (32.8)	
Sex				
Male	104	61 (58.7)	43 (41.3)	0.586
Female	16	11 (68.8)	5 (31.2)	
Primary tumor status (pT)				
pT1	9	6 (66.7)	3 (33.3)	0.025*
pT2	38	25 (65.8)	13 (34.2)	
pT3	35	25 (71.4)	10 (28.6)	
pT4	34	13 (38.2)	21 (61.8)	
Lymph node involvement (pN)				
Absent	65	40 (61.5)	25 (38.5)	0.574
Present	52	29 (55.8)	23 (44.2)	
Distant metastasis (M)				
Absent	115	69 (60.0)	46 (40.0)	1.000
Present	4	2 (50.0)	2 (50.0)	
pTNM stage				
pStage I	7	5 (71.4)	2 (28.6)	0.015*
pStage II	26	18 (69.2)	8 (30.8)	
pStage III	35	26 (74.3)	9 (25.7)	
pStage IV	49	21 (42.9)	28 (57.1)	

*Significantly different by the chi-square (χ^2^) test.

*Abbreviations*: *CYP26A1*, cytochrome P450, family 26, subfamily A, polypeptide 1; *pTNM*, pathological tumor-node-metastasis.

## Discussion

In this study, we found that CYP26A1 was highly expressed in cervical neoplasia and HNC cases. Such observations were consistent with the accumulated evidence demonstrating elevated CYP26A1 expression in various types of cancer [14–16]. Since CYP26A1 has been shown to exert a possible oncogenic effect, it is plausible for CYP26A1 expression to be potentially involved in the development and/or progression of cervical neoplasia and HNC. In addition, the data suggested that elevated CYP26A1 expression might contribute to carcinogenesis of the cervix, head, and neck by causing a state of functional VAD. There is no direct evidence to support this possibility; however, such a hypothesis is based on a mechanistic link between VAD and the increased risk of various cancer types [17]. Consistently, a number of published studies have revealed that individuals with VAD may accumulate DNA damage at a higher frequency, possibly resulting in an increased cancer risk and incidence [19, 20].

It is well known that chronic HPV infection is a necessary etiologic factor in the development of cervical neoplasia [1]. HPV-encoded oncoproteins, such as E6 and E7, have been shown to play a major role in HPV-mediated cervical carcinogenesis [21]. Both E6 and E7 proteins have immortalizing activity via their respective interactions with the tumor suppressor proteins p53 and retinoblastoma (Rb). Such interactions lead to the rapid degradation of p53 and Rb via the ubiquitin pathway and subsequent loss of p53 and Rb function, which abrogates apoptosis and deregulates cell cycle progression. Consistently, p53 mutation is reported to be a rare event in cervical cancer [22]. Indeed, we found that p53 immunoreactivity was absent in 44 of 50 (88%) patients with cervical malignancy and not associated with CYP26A1 expression. Thus, it is likely that the lack of deregulated p53 signaling and CYP26A1 overexpression may have independent effects on the progression of cervical neoplasia.

Although our results clearly demonstrated an increased expression of CYP26A1 in cervical neoplasia and HNC, the underlying molecular mechanism remains to be clarified. Because of the significance of transcriptional complexity in the regulatory mechanism of CYP26A1, dysregulated signaling resulting from a multifactorial process involving various genetic alterations in carcinogenesis of the cervix, head, and neck might offer a possible explanation to the molecular mechanism [23]. The signaling pathways involved may modulate the activity of different types of nuclear transcription factors that have yet to be identified as CYP26A1 regulators. Alternatively, CYP26A1 overexpression might be directly associated with the cumulative alterations of aberrant signaling, including activation of proto-oncogenes and inactivation of multiple tumor suppressor genes [12]. Such a hypothesis is partially supported by CYP26A1 overexpression-mediated changes in gene-expression, suggesting that many genes that favor cell survival are modulated by CYP26A1 expression [13].

We also hypothesized that CYP26A1 overexpression might be directly associated with the carcinogenic scenario of the uterine cervix, head, and neck through its role in RA metabolism. In support of this hypothesis, emerging data suggest that HPV-induced cervical carcinogenesis is associated with reduced serum levels of RA [24]. Similarly, head and neck carcinogenesis has also been suggested to associate with reduced RA serum levels [25]. Tobacco smoking is known to be an apparent risk factor for HNC. Since certain ingredients in tobacco smoke have been shown to induce an altered retinoid signaling, there is a possible linkage between RA metabolism induced by CYP26A1 and elevated carcinogenic insults in the head and neck. Thus, it is not surprising that CYP26A1 upregulation results in VAD-associated consequences in the squamous mucosa of the cervix, head, and neck, eventually leading to an increased risk for cancer development and progression. However, all the above-mentioned speculations need to be confirmed in future studies.

The exact function and expression pattern of CYP26A1 in cancer have not been elucidated. In the present study, immunohistochemical analysis showed an interesting cellular localization pattern of CYP26A1 in cervical neoplasia and HNC with varying nuclear expressions. However, available bioinformatics results indicate no nuclear localization signals in the CYP26A1 promoter. Although the effects of nuclear or cytoplasmic CYP26A1 in cancer remain unknown, the altered location of CYP26A1 potentially associates with the regulation of unidentified signaling pathways. While both nuclear and cytoplasmic CYP26A1 might be involved in carcinogenesis of the cervix, head, and neck, cytoplasmic CYP26A1 seems to possess more distinct functions than nuclear CYP26A1 in these patients. Based on the strong cytoplasmic expression of CYP26A1 in HSIL and SCC cases and progressively increased expression of CYP26A1 in cervical squamous neoplasia, we speculated that cytoplasmic localization of CYP26A1 strongly promoted the progression of squamous neoplasia of the cervix, head, and neck.

SCC of the cervix, head, and neck is a heterogeneous disease with complex molecular abnormalities. To our knowledge, the present study is the first to demonstrate the upregulation of CYP26A1 in cervical carcinoma and its precursor lesions, and to report that CYP26A1 expression increases with the progression of cervical squamous neoplasia. In addition, we observed increased expression of CYP26A1 in HNC patients and its significant correlation with primary tumor growth. Taken together, our data suggested that the enhanced expression of CYP26A1 might contribute to squamous neoplasia of the cervix, head, and neck, possibly via VAD-associated consequences. Therefore, we hypothesized that CYP26A1 upregulation might be an unrecognized mechanism of development and/or progression during squamous neoplasia of the cervix, head, and neck.

The present study clearly provided evidence of elevated CYP26A1 expression in cervical squamous neoplasia and HNC; however, the following issues remain to be clarified in future studies: 1) the exact role of CYP26A1 and its molecular impact on carcinogenesis, 2) the underlying regulatory mechanism of increased CYP26A1 expression in human cancers, 3) the function and expression pattern of cytoplasmic or nuclear CYP26A1 and the potential association between CYP26A1 cellular localization and various signaling pathways, and 4) the possible association between CYP26A1 overexpression and independent clinicopathological factors in different cancer types. Therefore, future studies are warranted to better understand the regulatory mechanism of CYP26A1 overexpression and its molecular impact on the neoplasia of human malignancy in the cervix, head, and neck.

## Conclusions

In the present study, we found that CYP26A1 expression was elevated in malignant and precancerous dysplastic lesions of the human cervix and increased with the progression of cervical squamous neoplasia. We also successfully demonstrated the elevated CYP26A1 expression in HNC patients and its significant correlation with primary tumor growth. Our data suggested that the enhanced expression of CYP26A1 might contribute to the development and progression of cervical malignancies and squamous neoplasia of the head and neck.

## Electronic supplementary material

Additional file 1: Table S1: CYP26A1 expression and the clinical profiles of cases with intraepithelial neoplasia of the cervix. (PDF 57 KB)

Additional file 2: Table S2: CYP26A1 expression and the clinical profiles of the cervical cancer tissue microarray. (PDF 65 KB)

Additional file 3: Table S3: CYP26A1 expression and the clinical profiles of the US Biomax laryngeal and pharyngeal cancer tissue microarray. (PDF 67 KB)

Additional file 4: Table S4: CYP26A1 expression and the clinical profiles of the Super Bio Chips multiple head and neck carcinoma tissue microarray. (PDF 61 KB)
