# Supplementary material for: Increased expression of the retinoic acid-metabolizing enzyme CYP26A1 during the progression of cervical squamous neoplasia and head and neck cancer
Source: BMC Res Notes. 2014 Oct 7;7:697. doi: 10.1186/1756-0500-7-697 (PMC4198729; doi:10.1186/1756-0500-7-697)
Supplement: Supplementary file 1 — Additional file 1: Table S1: CYP26A1 expression and the clinical profiles of cases with intraepithelial neoplasia of the cervix. (PDF 57 KB) [file 13104_2014_3230_MOESM1_ESM.pdf]

Table S1 (supplementary). CYP26A1 expression and the clinical profiles of cases with intraepithelial neoplasia of the cervix

No.	Age	Sex	Organ	Classification systems			CYP26A1 expression (%)
				Dysplasia/CIS[a]	CIN[b]	SIL[c]	
1	35	F	Cervix	Mild dysplasia	CIN1	LSIL	4
2	28	F	Cervix	Mild dysplasia	CIN1	LSIL	6
3	35	F	Cervix	Mild dysplasia	CIN1	LSIL	3
4	39	F	Cervix	Mild dysplasia	CIN1	LSIL	6
5	44	F	Cervix	Mild dysplasia	CIN1	LSIL	5
6	68	F	Cervix	Mild dysplasia	CIN1	LSIL	2
7	33	F	Cervix	Mild dysplasia	CIN1	LSIL	5
8	73	F	Cervix	Mild dysplasia	CIN1	LSIL	2
9	44	F	Cervix	Mild dysplasia	CIN1	LSIL	4
10	32	F	Cervix	Mild dysplasia	CIN1	LSIL	6
11	35	F	Cervix	Mild dysplasia	CIN1	LSIL	8
12	39	F	Cervix	Mild dysplasia	CIN1	LSIL	4

13	41	F	Cervix	Mild dysplasia	CIN1	LSIL	5
14	19	F	Cervix	Mild dysplasia	CIN1	LSIL	5
15	34	F	Cervix	Moderate dysplasia	CIN2	HSIL	9
16	36	F	Cervix	Moderate dysplasia	CIN2	HSIL	12
17	65	F	Cervix	Moderate dysplasia	CIN2	HSIL	7
18	39	F	Cervix	Moderate dysplasia	CIN2	HSIL	14
19	34	F	Cervix	Moderate dysplasia	CIN2	HSIL	11
20	40	F	Cervix	Moderate dysplasia	CIN2	HSIL	12
21	25	F	Cervix	Moderate dysplasia	CIN2	HSIL	18
22	44	F	Cervix	Moderate dysplasia	CIN2	HSIL	14
23	28	F	Cervix	Moderate dysplasia	CIN2	HSIL	16
24	35	F	Cervix	Moderate dysplasia	CIN2	HSIL	12
25	76	F	Cervix	Moderate dysplasia	CIN2	HSIL	8
26	34	F	Cervix	Moderate dysplasia	CIN2	HSIL	11
27	35	F	Cervix	Moderate dysplasia	CIN2	HSIL	17
28	34	F	Cervix	Severe dysplasia	CIN3	HSIL	18
29	45	F	Cervix	Severe dysplasia	CIN3	HSIL	26
30	46	F	Cervix	Severe dysplasia	CIN3	HSIL	25

31	43	F	Cervix	Severe dysplasia	CIN3	HSIL	28
32	35	F	Cervix	Severe dysplasia	CIN3	HSIL	16
33	56	F	Cervix	Severe dysplasia	CIN3	HSIL	21
34	52	F	Cervix	Severe dysplasia	CIN3	HSIL	26
35	40	F	Cervix	Severe dysplasia	CIN3	HSIL	15
36	40	F	Cervix	Severe dysplasia	CIN3	HSIL	32
37	64	F	Cervix	Severe dysplasia	CIN3	HSIL	26
38	29	F	Cervix	Severe dysplasia	CIN3	HSIL	33
39	36	F	Cervix	Severe dysplasia	CIN3	HSIL	31
40	52	F	Cervix	Severe dysplasia	CIN3	HSIL	25
41	30	F	Cervix	Severe dysplasia	CIN3	HSIL	36
42	52	F	Cervix	Carcinoma in situ	CIN3	HSIL	46
43	52	F	Cervix	Carcinoma in situ	CIN3	HSIL	57
44	40	F	Cervix	Carcinoma in situ	CIN3	HSIL	38
45	58	F	Cervix	Carcinoma in situ	CIN3	HSIL	42
46	79	F	Cervix	Carcinoma in situ	CIN3	HSIL	35
47	33	F	Cervix	Carcinoma in situ	CIN3	HSIL	64
48	60	F	Cervix	Carcinoma in situ	CIN3	HSIL	56
49	31	F	Cervix	Carcinoma in situ	CIN3	HSIL	51

50	53	F	Cervix	Carcinoma in situ	CIN3	HSIL	48
51	58	F	Cervix	Carcinoma in situ	CIN3	HSIL	57
52	36	F	Cervix	Carcinoma in situ	CIN3	HSIL	42
54	35	F	Cervix	Normal epithelium, match to #1			0
55	28	F	Cervix	Normal epithelium, match to #2			0
56	35	F	Cervix	Normal epithelium, match to #3			0
57	39	F	Cervix	Normal epithelium, match to #4			0
58	44	F	Cervix	Normal epithelium, match to #5			0
59	68	F	Cervix	Normal epithelium, match to #6			0
60	33	F	Cervix	Normal epithelium, match to #7			0

Abbreviations: [a] CIS, carcinoma in situ; [b] CIN, cervical intraepithelial neoplasia; [c] SIL, squamous intraepithelial lesion.
