# Supplementary material for: Increased expression of the retinoic acid-metabolizing enzyme CYP26A1 during the progression of cervical squamous neoplasia and head and neck cancer
Source: BMC Res Notes. 2014 Oct 7;7:697. doi: 10.1186/1756-0500-7-697 (PMC4198729; doi:10.1186/1756-0500-7-697)
Supplement: Supplementary file 2 — Additional file 2: Table S2: CYP26A1 expression and the clinical profiles of the cervical cancer tissue microarray. (PDF 65 KB) [file 13104_2014_3230_MOESM2_ESM.pdf]

Table S2 (supplementary). CYP26A1 expression and the clinical profiles of the cervical cancer tissue microarray

No.	Age	Sex	Organ	Diagnosis	pTNM	Stage	Follow-up months	Follow-up results	Cause of death	CYP26A1 (Score)	p53	Ki67 labeling index (%)
1	70	F	Cervix	Squamous cell carcinoma, microinvasive	T1a1N0M0	IA1	176	dead	Chronic renal failure	1	-	5
2	46	F	Cervix	Squamous cell carcinoma, microinvasive	T1a1N0M0	IA1	191	alive		0	-	< 5
3	42	F	Cervix	Squamous cell carcinoma, microinvasive	T1a1N0M0	IA1	191	alive		1	-	10
4	57	F	Cervix	Squamous cell carcinoma in situ	TisN0M0	0	189	alive		0	+	< 5
5	33	F	Cervix	Squamous cell carcinoma, microinvasive	T1a1N0M0	IA1	187	alive		1	-	5
6	35	F	Cervix	Squamous cell carcinoma in situ	TisN0M0	0	81	dead	Cancer	2	-	10
7	48	F	Cervix	Squamous cell carcinoma, microinvasive	T1a1N0M0	IA1	185	alive		0	-	< 5
8	39	F	Cervix	Squamous cell carcinoma, microinvasive	T1a1N0M0	IA1	183	alive		1	-	15
9	58	F	Cervix	Squamous cell carcinoma, microinvasive	T1a1N0M0	IA1	178	dead	Parkinson's disease	0	-	10
10	53	F	Cervix	Squamous cell carcinoma in situ	TisN0M0	0	181	alive		1	+	15
11	53	F	Cervix	Squamous cell carcinoma	T1b2N0M0	IB2	180	alive		1	-	< 5
12	53	F	Cervix	Squamous cell carcinoma	T2bN1M0	IIIB	20	alive		0	-	20
13	50	F	Cervix	Adenocarcinoma	T1b2N1M0	IIIB	103	dead	Cancer	0	-	15
14	45	F	Cervix	Squamous cell carcinoma	T1b2N1M0	IIIB	0	lost		2	-	10
15	49	F	Cervix	Squamous cell carcinoma	T1b1N1M0	IIIB	0	lost		2	-	25
16	35	F	Cervix	Squamous cell carcinoma	T1b1N0M0	IB1	0	lost		1	-	30
17	57	F	Cervix	Squamous cell carcinoma	T2a1N0M0	IIA1	3	lost		0	+	10
18	39	F	Cervix	Squamous cell carcinoma	T1b1N0M0	IB1	54	dead	Cancer	3	-	25
19	66	F	Cervix	Squamous cell carcinoma	T2a1N1M0	IIIB	190	dead	Lung cancer	2	-	10
20	55	F	Cervix	Squamous cell carcinoma	T1b2N0M0	IB2	36	dead	Cancer	3	-	15
21	64	F	Cervix	Squamous cell carcinoma	T1b2N1M0	IIIB	131	dead	Lung cancer	2	-	10
22	50	F	Cervix	Squamous cell carcinoma	T1b1N0M0	IB1	18	dead	Cancer	1	-	25
23	48	F	Cervix	Squamous cell carcinoma	T1b1N1M0	IIIB	165	alive		3	-	25
24	51	F	Cervix	Squamous cell carcinoma	T1b2N1M0	IIIB	56	dead	Cancer	2	-	15
25	54	F	Cervix	Squamous cell carcinoma	T1b1N0M0	IB1	164	alive		0	-	5
26	39	F	Cervix	Squamous cell carcinoma	T1b1N0M0	IB1	3	lost		2	-	5
27	58	F	Cervix	Squamous cell carcinoma	T1b1N0M0	IB1	0	lost		1	-	10
28	57	F	Cervix	Squamous cell carcinoma	T1b1N0M0	IB1	164	alive		0	+	< 5
29	55	F	Cervix	Squamous cell carcinoma	T1b1N0M0	IB1	0	lost		1	-	10
30	35	F	Cervix	Squamous cell carcinoma	T1b1N1M0	IIIB	162	alive		3	-	30
31	62	F	Cervix	Squamous cell carcinoma	T1b1N0M0	IB1	161	alive		1	-	15
32	64	F	Cervix	Squamous cell carcinoma	T1b2N1M0	IIIB	161	alive		0	+	10
33	60	F	Cervix	Adenosquamous carcinoma	T1b1N0M0	IB1	156	dead	Pancreas cancer	1	-	< 5
34	43	F	Cervix	Squamous cell carcinoma	T1b1N1M0	IIIB	160	alive		3	-	30
35	68	F	Cervix	Squamous cell carcinoma	T1b1N0M0	IB1	160	alive		1	-	15

36	45	F	Cervix	Squamous cell carcinoma	T1b2N0M0	IB2	160	alive		3	-	15
37	40	F	Cervix	Squamous cell carcinoma	T1b1N0M0	IB1	159	alive		2	-	25
38	58	F	Cervix	Adenosquamous carcinoma	T1b1N0M0	IB1	158	alive		0	+	10
39	67	F	Cervix	Squamous cell carcinoma	T1b1N0M0	IB1	157	alive		2	-	25
40	48	F	Cervix	Squamous cell carcinoma	T1b1N1M0	IIIB	157	alive		2	-	15
41	62	F	Cervix	Squamous cell carcinoma	T1b2N1M0	IIIB	0	dead	Thrombosis	0	-	30
42	32	F	Cervix	Squamous cell carcinoma	T1b1N1M0	IIIB	17	dead	Cancer	0	-	20
43	55	F	Cervix	Squamous cell carcinoma	T2a1N1M0	IIIB	7	lost		0	-	15
44	61	F	Cervix	Squamous cell carcinoma	T2bN1M0	IIIB	2	lost		1	-	10
45	34	F	Cervix	Adenocarcinoma	T1b2N1M0	IIIB	20	dead	Cancer	1	-	10
46	53	F	Cervix	Squamous cell carcinoma	T1b1N0M0	IB1	176	alive		2	-	20
47	45	F	Cervix	Squamous cell carcinoma	T1b1N0M0	IB1	165	alive		1	-	< 5
48	65	F	Cervix	Squamous cell carcinoma	T1b1N0M0	IB1	133	dead	Cerebrovascular disease	1	-	20
49	47	F	Cervix	Squamous cell carcinoma	T1b1N1M0	IIIB	162	alive		2	-	25
50	59	F	Cervix	Adenosquamous carcinoma	T1b2N1M0	IIIB	156	alive		3	-	10
51	62	F	Lymph node	Metastatic carcinoma of N0.41						0		
52	32	F	Lymph node	Metastatic carcinoma of N0.42						1		
53	55	F	Lymph node	Metastatic carcinoma of N0.43						1		
54	61	F	Lymph node	Metastatic carcinoma of N0.44						1		
55	34	F	Lymph node	Metastatic carcinoma of N0.45						0		
56	53	F	Cervix	Normal of No.46						0		
57	45	F	Cervix	Normal of No.47						0		
58	65	F	Cervix	Normal of No.48						0		
59	47	F	Cervix	Normal of No.49						0		
60		F	Blank	Carbon								

Abbreviation: pTNM, pathological tumor-node-metastasis
