# Supplementary material for: Increased expression of the retinoic acid-metabolizing enzyme CYP26A1 during the progression of cervical squamous neoplasia and head and neck cancer
Source: BMC Res Notes. 2014 Oct 7;7:697. doi: 10.1186/1756-0500-7-697 (PMC4198729; doi:10.1186/1756-0500-7-697)
Supplement: Supplementary file 3 — Additional file 3: Table S3: CYP26A1 expression and the clinical profiles of the US Biomax laryngeal and pharyngeal cancer tissue microarray. (PDF 67 KB) [file 13104_2014_3230_MOESM3_ESM.pdf]

Table S3 (supplementary). CYP26A1 expression and the clinical profiles of the US Biomax laryngeal and pharyngeal cancer tissue microarray

No.	Age	Sex	Organ	Diagnosis	Grade*	pTNM	Stage	Intensity score	Proportion score	Total score	Note
1	61	F	Palate	Squamous cell carcinoma of palate	1	T1N0M0	I	0	0	0	
2	61	M	Lower lip	Squamous cell carcinoma of lower lip	1	T1N0M0	I	0	0	0	
3	77	M	Lower lip	Squamous cell carcinoma of lower lip	1	T1N0M0	I	1	1	2	
4	75	F	Upper jaw	Squamous cell carcinoma of upper jaw	N/A	T1N0M0	I	0	0	0	
5	55	M	Tongue	Squamous cell carcinoma of tongue	1	T1N0M0	I	0	0	0	
6	67	M	Palate	Squamous cell carcinoma of right palate	1	T2N0M0	II	0	0	0	
7	67	M	Larynx	Squamous cell carcinoma of larynx	1	T2N0M0	II	1	1	2	
8	70	M	Maxillary sinus	Squamous cell carcinoma of right maxillary sinus	1	T2N0M0	II	2	2	4	
9	40	M	Larynx	Squamous cell carcinoma of larynx	2	T2N1M1	IV	3	2	5	Nuclear staining
10	55	M	Epiglottis	Squamous cell carcinoma of epiglottis	2	T4N0M0	IV	2	2	4	
11	57	M	Pharynx	Squamous cell carcinoma of pharynx	2	T4N0M0	IV	3	2	5	
12	49	M	Gingiva	Squamous cell carcinoma of left gingiva	1	T4N0M0	IV	1	1	2	
13	50	M	Tongue	Squamous cell carcinoma of root of tongue	2	T3N0M0	III	0	0	0	
14	54	M	Upper jaw	Squamous cell carcinoma of left upper jaw	2	T3N0M0	III	0	0	0	
15	72	M	Larynx	Squamous cell carcinoma of larynx	2	T2N0M0	II	0	0	0	
16	50	M	Tongue	Squamous cell carcinoma of tongue	N/A	T2N0M0	II	0	0	0	
17	74	M	Nose	Squamous cell carcinoma of nasal root	2	T2N0M0	II	0	0	0	
18	49	M	Larynx	Squamous cell carcinoma of larynx	2	T4N1M0	IV	3	3	6	Nuclear staining
19	50	M	Larynx	Squamous cell carcinoma of larynx	2	T2N1M0	III	1	1	2	
20	90	M	Cheek	Squamous cell carcinoma of cheek	2	T2N0M0	II	2	2	4	Nuclear staining
21	67	M	Larynx	Squamous cell carcinoma of larynx	2	T3N1M0	III	1	1	2	
22	56	M	Epiglottis	Squamous cell carcinoma of epiglottis	2	T4N0M0	IV	3	2	5	
23	66	M	Laryngeal pharynx	Squamous cell carcinoma of left laryngeal pharynx	2	T3N2M0	IV	2	2	4	
24	58	M	Larynx	Squamous cell carcinoma of larynx	2	T3N1M1	IV	3	3	6	
25	60	M	Tongue	Squamous cell carcinoma of tongue	3	T2N0M0	II	0	0	0	
26	67	M	Pyriform sinus	Squamous cell carcinoma of pyriform sinus	3	T2N0M0	II	1	1	2	
27	56	F	Nasopharynx	Squamous cell carcinoma of nasopharynx	3	T2N0M0	II	1	0	1	
28	48	M	Submaxilla	Squamous cell carcinoma of left submaxilla	1	T2N0M0	II	3	2	5	
29	56	M	Laryngeal pharynx	Squamous cell carcinoma of laryngeal pharynx	2	T2N0M0	II	1	1	2	
30	43	M	Submaxilla	Squamous cell carcinoma of right submaxilla	2	T2N0M0	II	0	0	0	
31	70	M	Submaxilla	Squamous cell carcinoma of gingiva of right submaxilla	2	T2N0M0	II	2	2	4	
32	61	F	Submaxilla	Squamous cell carcinoma of left submaxilla	2	T2N0M0	II	2	2	4	
33	56	F	Oral cavity	Squamous cell carcinoma of mouse floor	2	T2N0M0	II	0	0	0	
34	55	M	Larynx	Squamous cell carcinoma of larynx	2	T3N1M0	III	1	1	2	
35	50	M	Larynx	Squamous cell carcinoma of larynx	2	T3N1M0	III	1	1	2	
36	49	M	Larynx	Squamous cell carcinoma of larynx	3	T2N1M0	III	0	0	0	
37	71	M	Epiglottis	Squamous cell carcinoma of epiglottis	2	T3N1M0	III	1	1	2	
38	50	F	Laryngeal pharynx	Squamous cell carcinoma of laryngeal pharynx	2	T2N1M0	III	2	2	4	
39	58	M	Pyriform sinus	Squamous cell carcinoma of pyriform sinus	2	T3N0M0	III	3	3	6	
40	71	M	Laryngeal pharynx	Squamous cell carcinoma of laryngeal pharynx	2	T2N1M0	III	1	1	2	
41	61	M	Epiglottis	Squamous cell carcinoma of epiglottis	2	T2N1M0	III	2	2	4	Nuclear staining

42	44	M	Laryngeal pharynx	Squamous cell carcinoma of laryngeal pharynx	2	T2N1M0	III	2	1	3	Nuclear staining
43	61	M	Larynx	Squamous cell carcinoma of larynx	2	T3N0M0	III	0	0	0	
44	45	M	Cheek	Squamous cell carcinoma of right cheek	3	T2N0M0	II	0	0	0	
45	47	M	Larynx	Squamous cell carcinoma of larynx	2	T2N0M0	II	2	2	4	Nuclear staining
46	63	M	Epiglottis	Squamous cell carcinoma of epiglottis	2	T3N1M0	III	2	2	4	
47	61	M	Epiglottis	Squamous cell carcinoma of epiglottis	3	T2N0M0	II	0	0	0	
48	75	M	Maxillary sinus	Squamous cell carcinoma of maxillary sinus	3	T4N0M0	IV	1	1	2	
49	50	M	Pyriform sinus	Squamous cell carcinoma of pyriform sinus	3	T2N0M0	II	2	3	5	
50	64	M	Larynx	Squamous cell carcinoma of larynx	3	T2N0M0	II	0	0	0	
51	38	F	Lower lip	Squamous cell carcinoma of lower lip	N/A	T2N0M0	II	0	0	0	
52	56	F	Cheek	Squamous cell carcinoma of cheek	3	T3N0M0	III	0	0	0	
53	42	F	Nasal sinus	Squamous cell carcinoma of nasal sinus	N/A	T3N0M0	III	1	1	2	
54	47	M	Larynx	Squamous cell carcinoma of larynx	N/A	T2N1M0	III	0	0	0	
55	51	M	Larynx	Squamous cell carcinoma of larynx	2	T2N1M0	III	2	2	4	
56	38	M	Pyriform sinus	Squamous cell carcinoma of pyriform sinus	N/A	T2N2M0	IV	2	2	4	
57	74	M	Laryngeal pharynx	Squamous cell carcinoma of laryngeal pharynx	3	T4N0M0	IV	3	3	6	Nuclear staining
58	48	M	Laryngeal pharynx	Squamous cell carcinoma of laryngeal pharynx	2	T4N0M0	IV	3	3	6	Nuclear staining
59	65	M	Larynx	Squamous cell carcinoma of larynx	3	T3N2M0	IV	1	1	2	
60	75	M	Nasal sinus	Squamous cell carcinoma of nasal sinus	3	T4N0M0	IV	2	2	4	
61	32	F	Maxillary sinus	Squamous cell carcinoma of maxillary sinus	N/A	T2N0M0	II	0	0	0	
62	40	F	Lymph node	Metastatic mucoepidermoid carcinoma of the neck	3			3	3	6	Nuclear staining
63	67	M	Lymph node	Metastatic mucoepidermoid carcinoma of mouth floor	3			0	0	0	
64	55	M	Lymph node	Metastatic squamous cell carcinoma of left upper gingiva	2			3	3	6	Nuclear staining
65	47	M	Lymph node	Metastatic squamous cell carcinoma of oral cavity	1			1	1	2	
66	49	M	Lymph node	Metastatic squamous cell carcinoma of hypoglottis	2			2	2	4	
67	53	M	Lymph node	Metastatic squamous cell carcinoma of laryngeal pharynx	3			3	3	6	Nuclear staining
68	43	M	Lymph node	Metastatic acinic cell carcinoma of the neck	3			0	0	0	
69	32	F	Lymph node	Metastatic acinic cell carcinoma of the neck	1			0	0	0	
70	38	M	Tongue	Cancer adjacent normal tongue tissue				0	0	0	
71	28	M	Tongue	Normal tongue tissue				0	0	0	
72	27	F	Tongue	Normal tongue tissue				0	0	0	
73	48	M	Tongue	Normal tongue tissue				0	0	0	
74	42	F	Tongue	Normal tongue tissue				0	0	0	
75	15	F	Tongue	Normal tongue tissue				0	0	0	
76	19	F	Tongue	Normal tongue tissue				0	0	0	
77	35	M	Tongue	Normal tongue tissue				0	0	0	
78	18	F	Tongue	Normal tongue tissue				0	0	0	
79	19	F	Tongue	Normal tongue tissue				0	0	0	
80	28	M	Pharynx	Normal pharynx tissue				0	0	0	
81	55	F	Liver	Hepatocellular liver cancer	3	T3N0M0	N/A	3	3	6	

*The grade 1-3 is equivalent to well-differentiated, moderately-differentiated, or poorly differentiated, respectively, under microscope.
Abbreviation: pTNM, pathological tumor-node-metastasis
