# Supplementary material for: Increased expression of the retinoic acid-metabolizing enzyme CYP26A1 during the progression of cervical squamous neoplasia and head and neck cancer
Source: BMC Res Notes. 2014 Oct 7;7:697. doi: 10.1186/1756-0500-7-697 (PMC4198729; doi:10.1186/1756-0500-7-697)
Supplement: Supplementary file 4 — Additional file 4: Table S4: CYP26A1 expression and the clinical profiles of the Super Bio Chips multiple head and neck carcinoma tissue microarray. (PDF 61 KB) [file 13104_2014_3230_MOESM4_ESM.pdf]

Table S4 (supplementary). CYP26A1 expression and the clinical profiles of the Super Bio Chips multiple head and neck carcinoma tissue microarray

No.	Age	Sex	Organ	Diagnosis	Grade*	pTNM	Stage	Intensity score	Proportion score	Total score	Note
1	43	M	Larynx, pyriform sinus	Squamous cell carcinoma	2	T4N3M0	IVB	0	0	0	
2	55	M	Larynx, subglottic	Squamous cell carcinoma	2	T4N0M0	IVA	1	1	2	
3	72	M	Larynx, supraglottic	Squamous cell carcinoma	2	T3N0M0	III	3	2	5	Nuclear staining
4	63	M	Esophagus	Squamous cell carcinoma, basaloid type	N/A	T3N0M0	III	1	0	1	
5	67	M	Larynx, transglottic	Squamous cell carcinoma	1	T4N2M0	IVA	2	0	2	
6	63	M	Larynx, pyriform sinus	Squamous cell carcinoma	2	T3N2M0	IVA	2	1	3	
7	60	M	Larynx, supraglottic	Squamous cell carcinoma	2	T4N2M0	IVA	1	1	2	
8	61	M	Larynx, glottic	Squamous cell carcinoma	2	T4N0M0	IVA	1	1	2	
9	66	M	Larynx, subglottic	Squamous cell carcinoma	2	T4N2M0	IVA	3	2	5	
10	56	M	Larynx, glottic	Squamous cell carcinoma	1	T1N0M0	I	2	1	3	
11	62	M	Larynx, supraglottic	Squamous cell carcinoma	2	T3N2M0	IVA	3	1	4	Nuclear staining
12	56	M	Larynx, pyriform sinus	Squamous cell carcinoma	2	T4N2M0	IVA	2	1	3	
13	54	M	Larynx, supraglottic	Squamous cell carcinoma	1	T3N0M0	III	2	0	2	
14	48	M	Larynx, transglottic	Squamous cell carcinoma	1	T3N0M0	III	3	2	5	
15	61	M	Larynx, supraglottic	Squamous cell carcinoma	1	T3N0M0	III	0	0	0	
16	66	M	Soft tissue, neck	Squamous cell carcinoma, from hypopharynx	1	T4N0M0	IVA	2	1	3	
17	51	M	Larynx, supraglottic	Squamous cell carcinoma	2	T4N2M0	IVA	0	0	0	
18	46	M	Soft tissue, neck	Squamous cell carcinoma, from hypopharynx	N/A	T3N2M0	IVA	2	1	3	Nuclear staining
19	52	M	Larynx, supraglottic	Squamous cell carcinoma	2	T3N0M0	III	1	1	2	
20	52	F	Nasal cavity	Undifferentiated carcinoma	4	T4NxM0	IVA	0	0	0	
21	64	M	Larynx, transglottic	Squamous cell carcinoma, basaloid type	N/A	T3N2M0	IVA	0	0	0	
22	28	F	Lymph node	Metastatic undifferentiated carcinoma (from nasopharynx)	4	T1N1M0	II	2	1	3	
23	63	M	Nasopharynx	Undifferentiated carcinoma	4	TxN2M1	IVC	0	0	0	
24	77	M	Neck	Undifferentiated carcinoma	4	TxN1M0	N/A	1	0	0	
25	51	M	Larynx, transglottic	Squamous cell carcinoma	1	T4N2M0	IVA	2	1	3	
26	67	F	Neck	Squamous cell carcinoma, from hypopharynx	2	TxN0M0	N/A	2	1	3	
27	59	M	Nasal cavity	Squamous cell carcinoma	3	T4N0M0	IVA	1	1	2	
28	55	M	Larynx, subglottic	Squamous cell carcinoma	2	T3N0M0	III	2	1	3	
29	60	M	Larynx, transglottic	Squamous cell carcinoma	1	T4N0M0	IVA	2	1	3	
30	70	M	Oropharynx	Undifferentiated carcinoma	4	T1N2M0	IVA	1	0	1	
31	55	M	Hypopharynx	Squamous cell carcinoma	3	T4N1M0	IVA	2	1	3	Nuclear staining
32	62	M	Larynx, pyriform sinus	Squamous cell carcinoma	N/A	T3N0M0	III	0	0	0	
33	48	M	Soft tissue, neck	Squamous cell carcinoma (from larynx)	N/A	T2N1M0	III	1	1	2	
34	66	M	Larynx, supraglottic	Squamous cell carcinoma	2	T4N2M0	IVA	2	1	3	
35	58	M	Larynx, subglottic	Squamous cell carcinoma	1	T4N0M0	IVA	3	2	5	Nuclear staining
36	76	M	Larynx, supraglottic	Squamous cell carcinoma	2	T3N1M0	III	1	1	2	
37	61	M	Larynx, supraglottic	Squamous cell carcinoma	1	T4N2M0	IVA	3	2	5	
38	66	M	Submandibular gland	Undifferentiated carcinoma	4	T3N2M0	III	1	0	1	
39	54	M	Tonsil	Squamous cell carcinoma	3	T2N2M0	IVA	1	0	1	
40	61	M	Nose	Squamous cell carcinoma	3	T3N0M0	III	1	0	1	
41	65	M	Larynx, glottic	Squamous cell carcinoma	2	T3NxM1	IVC	0	0	0	

42	54	M	Skin, neck	Undifferentiated carcinoma, from nasopharynx	4	T4N2M0	IVA	2	1	3	
43	82	F	Larynx, supraglottic	Squamous cell carcinoma	2	T3N1M0	III	0	0	0	
44	56	M	Larynx, subglottic	Squamous cell carcinoma	N/A	T4N2M0	IVA	1	0	1	
45	75	M	Larynx, transglottic	Squamous cell carcinoma	1	T4N0M0	IVA	3	2	5	Nuclear staining
46	69	M	Larynx, glottic	Squamous cell carcinoma	3	T4N2M0	IVA	3	1	4	
47	43	M	Larynx, subglottic	Squamous cell carcinoma	2	T3N0M0	III	1	0	1	
48	60	M	Larynx, glottic	Squamous cell carcinoma	N/A	T4N0M0	IVA	3	3	6	
49	66	M	Larynx, supraglottic	Squamous cell carcinoma	3	T2N0M0	II	1	0	1	
50	52	M	Larynx, supraglottic	Squamous cell carcinoma	N/A	T3N1M0	III	1	0	1	
51	58	M	Larynx, pyriform sinus	Squamous cell carcinoma	N/A	T4N2M0	IVA	3	1	4	
52	47	F	Maxilla	Undifferentiated carcinoma	4	T1N0M0	I	3	1	4	Nuclear staining
53	65	M	Larynx, glottic	Squamous cell carcinoma	1	T4N2M0	IVA	1	1	2	
54	78	M	Larynx, supraglottic	Squamous cell carcinoma	2	T3N1M0	III	1	1	2	
55	69	M	Epiglottis	Squamous cell carcinoma, basaloid type	N/A	T2N0M0	II	2	0	2	
56	60	M	Epiglottis	Squamous cell carcinoma	2	T2N2M0	IVA	1	1	2	
57	46	F	Lymph node, neck	Metastatic undifferentiated carcinoma (unknown primary site)	4	N/A	N/A	1	1	2	
58	70	M	Larynx, glottic	Squamous cell carcinoma	3	T4N2M0	IVA	0	0	0	
59	79	M	Larynx, supraglottic	Squamous cell carcinoma	3	T3N2M0	IVA	1	0	1	
60	Blank		Carbon								

*The grade 1-4 is equivalent to well-differentiated, moderately-differentiated, poorly-differentiated, or undifferentiated, respectively, under microscope.
Abbreviation: pTNM, pathological tumor-node-metastasis
